# Development of Novel DNA Cleavage Systems Based on Copper Complexes. Synthesis and Characterisation of Cu(II) Complexes of Hydroxyflavones

**DOI:** 10.1155/MBD.2000.365

**Published:** 2000

**Authors:** F. Ben-Allal el Amrani, L. Perelló, J. Borrás, L. Torres

**Affiliations:** 1 Department of Inorganic Chemistry University of Valencia Faculty of Pharmacy Vicent Andrés Estellés s/n Burjassot (Valencia) E- 46100 Spain; 2 Department of Biochemistry University of Valencia Faculty of Pharmacy Vicent Andrés Estellés s/n Burjassot (Valencia) E- 46100 Spain

## Abstract

Copper(II) complexes of several hydroxyflavones were prepared and characterised through their physico-chemical properties. The nuclease activity of three synthesised complexes is reported. These copper(II) complexes present more nuclease activity than the ligands and the copper(II) ion.


### DEVELOPMENT OF NOVEL DNA CLEAVAGE SYSTEMS BASED ON COPPER COMPLEXES. SYNTHESIS AND CHARACTERISATION OF Cu(II) COMPLEXES OF HYDROXYFLAVONES

F. Ben-Allal el Amrani[1], L. Perelló[1], J. Borrás*[1] and L. Torres[2]

[1]Department of Inorganic Chemistry [2]Department of Biochemistry
University of Valencia, Faculty of Pharmacy, Vicent Andrés Estellés s/n, E- 46100, Burjassot (Valencia) Spain <Joaquin.Borras@uv.es>

**Abstract** : Copper(II) complexes of several hydroxyflavones were prepared and characterised through their physico-chemical properties. The nuclease activity of three synthesised complexes is reported. These copper(II) complexes present more nuclease activity than the ligands and the copper(II) ion.

### INTRODUCTION

The flavonoids are a group of natural substances reported as being present in all higher plants.[1] The basic flavone structure consists of 1,4-benzopyrone with phenyl substitution in position 2. The different groups of flavonoids differ in the number and position of substitutents on the aromatic ring, and in the extent and character of the oxidation on the pyrone portion of the molecule. Through their hydroxyl groups, the flavonoids are able to combine with sugars to form glycosides. In this sense, over 4000 different species of flavonoids have been described in fruits and vegetables, seeds, stems and flowers. They are therefore important component of the human diet. On average, a mixed Western diet supplies about 1 g of flavonoids daily. The flavonoids exhibit a broad range of biochemical and pharmacological effects, some of which suggesting that a number of compounds of this group may affect the different cell systems. The flavonoids are probably the most common and active antioxidants in the human diet. In this sense, they are active both in hydrophilic and lipophilic systems. The mechanism responsible for the antioxidant activity appears to be dual. On one hand, they act as free radical scavengers, and on the other hand they are able to chelate metal ions, thereby reducing metal induced peroxidation.

Today it is known that the excessive production of free radicals and lipid peroxidation may be implicated in the development of certain chronic diseases such as atherosclerosis and cancer.

The quercetin (3',4',5,7-tetrahydroxyflavonol) and other 3-hydroxyflavones are degraded to depside and carbon monoxide by the quercetinase, a dioxygenase produced by the *Aspergillus flavus*.[2] Quercetinase is a Cu(II)-containing dioxygenase. A Cu(II) chelate of quercetin is the key intermediate in the enzymatic reaction. In order to determine if the mode of coordination of flavones may give information relevant to the enzyme-quercetine interaction, Speier et al[3-8] prepared copper(I) and copper (II) binary and ternary complexes of 3-hydroxyflavone, where the flavone interacts with the Cu(II) through the $O_{hydroxo}$ and the $O_{carbonyl}$ atoms. The synthesis and physicochemical properties of M(II) (Co, Ni and Cu) complexes with 5,7-dyhroxyflavone was reported.[9,10]

On the other hand, Hadi et al.[11,12] have shown that quercetin and other flavonoids cause strand scission in DNA in the presence of Cu(II) and that his reaction is associated with transient reduction of Cu(II) to Cu(I) and the generation of active species. Of the several metal ion tested [Cu(II), Fe(II), Fe(III), Co(II), Mn(II), Ni(II) and Ca(II)] only Cu(II) and Fe(III) complemented quercetin in the DNA breakage reaction. They proposed that the formation of a ternary complex of quercetin, DNA and Cu(II)/Cu(I) is involved in the generation of the oxygen active species that are the cause of the strand of DNA. A similar reaction dependent of metal ions and involving oxygen radicals has been reported with 1,10-phenantroline.[13]

DNA-directed sequence-specific reagents capable of controlled chemical cleavage may also serve as molecular biological tools, either in the sense of standard but artificial restriction endonucleases or for a larger scale genome mapping.

With this in mind, it is important to develop not only novel DNA sequence reading molecules, but also chemical cleavage systems which are easily amenable to chemical synthesis manipulation and have suitably biocompatibility (e.g. stability, cellular penetration, recycling).[14] Recently several papers about the nuclease activity of copper complexes were reported. [15-26]

Until now no paper concerning the nuclease activity of the copper complexes of flavones has been reported.

In the present paper we describe the synthesis and characterisation of a number of copper complexes of flavones (their structure is summarised in Figure 1) and the nuclease activity of three copper-flavone complexes. The antioxidant activity of these flavones were recently described.[27]

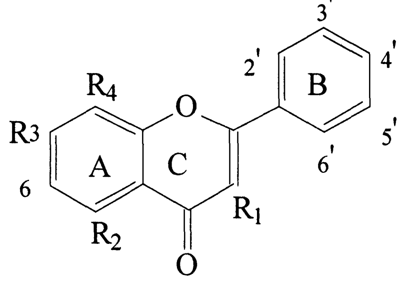

| | | |
|---|---|---|
| H3HF | $R_1$ = OH | $R_2 = R_3 = R_4$ = H |
| $H_2$5,7DHF | $R_1 = R_4$ = H | $R_2 = R_3$ = OH |
| $H_2$7,8DHF | $R_1 = R_2$ = H | $R_3 = R_4$ = OH |

Figure 1. Structure of hydroxyflavones

### MATERIALS AND METHODS

The flavonoids 3-hydroxyflavone (H3HF), 5,7-dihydroxyflavone ($H_2$5,7DHF), 7,8-dihydroxyflavone ($H_2$7,8DHF) were from Apin Chemicals Ltd. Cu(II) salts, hydrogen peroxide, ascorbic acid, bromophenol blue and ethidium bromide from Sigma Chemical Co. Plasmid pBR322 and agarose was purchased from Boehringer Manhein. Plasmid pUCI was a gift of the Departamento de Bioquimica de la Universitat de Valencia.

UV-Vis data were obtained on a Shidmazu 2101-PC instrument, while the infrared spectra were recorded as KBr disk on a Mattson Satellite Ftir spectrometer. X-band EPR spectra were collected at room temperature on a Bruker ER D200 spectrometer.

**Synthesis of complexes**

$Cu(3HF)_2$ ; $Cu(H5,7DHF)_2(MeOH)_2$ ; $Cu(7,8DHF)(H_2O)_2$.-

1 mmol of copper acetate was dissolved in 50 ml of MeOH. A different amount of H3HF, $H_2$5,7DHF and $H_2$7,8DHF according to molar ratios metal : flavone (Cu :H3HF = 1 : 1 ; Cu : $H_2$5,7DHF and Cu : $H_2$7,8DHF = 2 :1) was then added with stirring. After several hours solids were obtained, which were filtered and air-dried.

Anal. for $Cu(3HF)_2$ , Found : C 66.2 ; H 3.6 ; Cu 11.3 Calc. for $C_{30}H_{18}O_6Cu$ : C 66.9 ; H 3.4 ; Cu 11.8 ; IR bands : 1590 $cm^{-1}$ $\nu_{st}$(C=O) ; 1210 ; 810 $cm^{-1}$ $\nu$(-C-O-C-).

Anal. for $Cu(H5,7DHF)_2(MeOH)_2$, Found : C 60.4 ; H 4.0 ; Cu 9.7. Calc. for $C_{32}H_{26}O_{10}Cu$ : C 60.6 ; H 4.1 ; Cu 10.0 ; IR band : 1630 $cm^{-1}$ $\nu_{st}$(C=O) ; 1060 ; 840 $cm^{-1}$ $\nu$(-C-O-C-).

Anal. for $Cu(7,8DHF)(H_2O)_2$, Found : C 50.8 ; H 3.2 ; Cu 18.1 . Calc. for $C_{15}H_{12}O_6Cu$ : C 51.2 ; H 3.4 ; Cu 18.1. IR bands : 1620 $cm^{-1}$ $\nu_{st}$(C=O) ; 1080 ; 820 $cm^{-1}$ $\nu$(-C-O-C-)

Cu(7,8DHF)(MeOH)

To a 50 ml of a methanolic solution containing 1 mmol of $Cu(OCH_3)_2$, 1 mmol of $H_2$7,8DHF was added with continuous stirring. After an hour approximately it appears a brown solid which is filtered, washed with methanol and air-dried.

Anal. Found : C 54.9 ; H 3.2 ; Cu 17.7 ; Calc. for $C_{16}H_{12}O_5Cu$ : C 55.2 ; H 3.5 ; Cu 18.3. IR bands : 1620 $cm^{-1}$ $\nu_{st}$(C=O) ; 1080 ; 820 $cm^{-1}$ $\nu$(-C-O-C-).

$Cu(H5,7DHF)_2$ $(H_2O)_2$

To a 25 ml of a methanolic solution of $Cu(ClO_4)_2.6H_2O$, 1 mmol of the 5,7$H_2$DHF was added with stirring. After 30 min, 0.5 ml of butylamine (30%) was added. Immediately a yellow solid appeared, that was filtered, washed with methanol and air-dried until constant weight.

Anal. Found : C 59.2 ; H 3.6 ; Cu 10.6 ; Calc. for $C_{30}H_{22}O_{10}Cu$ : C 59.5 ; H 3.7 ; Cu 10.5. IR bands: 1620 $cm^{-1}$ $\nu_{st}$(C=O) ; 1080 ; 820 $cm^{-1}$ $\nu$(-C-O-C-).

$Cu(3HF)_2(NH_3)(H_2O)$ and $Cu(7,8DHF)(NH_3)_2(H_2O)_3$.-

To a 25 ml of a methanolic solution of $Cu(ClO_4)_2.6H_2O$, 1 mmol of the H3HF or $H_2$7,8DHF was added with stirring. After 30 min, 0.5 ml of concentrated $NH_3$ (30%) was added. Immediately yellow and brown solids respectively appeared, that were filtered, washed with methanol and air-dried until constant weight.

Anal for $Cu(3HF)_2(NH_3)(H_2O)$, found : C 62.8 ; H 3.8 ; N 2.4 ; Cu 11.4. Calc. for $C_{30}H_{23}NO_7Cu$ : C 62.9 ; H 4.0 ; N 2.4 ; Cu 11.1. IR bands: 3360, 3280 $cm^{-1}$ $\nu_{st}$(N-H) ; 1620 $cm^{-1}$ $\nu_{st}$(C=O) ; 1210; 890 $cm^{-1}$ $\nu$(-C-O-C-).

Anal. for $Cu(7,8DHF)(NH_3)_2(H_2O)_3$, found : C 43.8 ; H 4.9 ; N 6.4 ; Cu 16.0. Found for $C_{15}H_{20}N_2O_7Cu$ : C 44.6 ; H 5.0 ; N 6.9 ; Cu 15.7. IR bands : 3360, 3240 $cm^{-1}$ $\nu_{st}$(N-H) ; 1620 $cm^{-1}$ $\nu_{st}$(C=O) ; 1080 ; 810 $cm^{-1}$

ν(-C-O-C-)

**Cleavage of pBR322 and pUCI by Cu(II)-7,8DHF complexes**

A typical reaction was carried out by mixing 16 μl of the Cu(II) complex 0.625 μM in buffer Tris-HCl pH = 8, 2 μl of 0.25 μg/μl pBR322 or pUCI and 2 μl of $H_2O_2$ 500 μM. The resulting solution contains 0.5 μM of the complex, 0.025 μg/μl pBR322 or pUCI and 50 μM of $H_2O_2$. After allowing the sample to incubate at 25 C for a 30 min, 3 μl of a quench buffer solution consisting of a 0.2% bromophenol blue, 24% glycerol and 1mM edta, was added. Then the solution was subjected to electrophoresis on a 1% agarose gel in 1x TBE buffer (0.1M tris borate, 0.2 mM edta) at 80 V about 4 h. The gel was stained with 0.5 μg/ml ethidium bromide and photographed on a UV transilluminator with a Polaroid camera MP4 containing an Agfapan (negative/positive) film.

### RESULTS AND DISCUSSION

The band assigned to ν(O-H) of the H3HF disappears in the IR spectrum of the $Cu(3HF)_2$ complex. In the IR spectra of the rest of the synthesised complexes the existence of water or methanol molecules does not permit suggestions about the band attributed to ν(O-H).

A shift of 20 $cm^{-1}$ is observed for the band attributed to ν(C=O) in the IR spectra of the complexes with respect to the ligands.[5]

The ν(C-O-C) bands remain unchanged in the metal complexes of H3HF an $H_2$5,7DHF with respect to the IR spectra of the ligands. However a shift of these vibrations take place in the IR spectra of the metal complexes of $H_2$7,8DHF (1080 in the complexes and 1160 $cm^{-1}$ in the ligand). This is probably due to the deprotonation and coordination of the hydroxo groups at C7 and C8, neighbour of the C9-O-C10 group.

The complexes $Cu(3HF)_2(NH_3)(H_2O)$ and Cu(7,8 DHF)$(NH_3)_2(H_2O)_3$ show two peaks at 3360 and 3280 $cm^{-1}$ corresponding to ν(N-H) of ammonia.

According to the IR spectra it is possible to conclude that the $3HF^-$ interacts with the Cu (II) ion as a bidentate ligand through the $O_{carbonyl}$ and $O_{hydroxo}$ atoms in similarly as for the copper flavonolate complex.[7] Probably, the $H5,7DHF^-$ anion links to Cu(II) in a similar manner, while the $7,8DHF^{2-}$ anion must interact with Cu(II) through the contiguous deprotonated OH.

The electronic spectra of all the copper(II) complexes exhibit in the solid state one major absorption band in the 12500-20000 $cm^{-1}$ region. This band is attributed to d-d transitions in an octahedral geometry or a square pyramidal distorted structure. One band at 22500-24500 $cm^{-1}$ is attributed to a charge-transfer transition from the flavonolato ligand to the metal atom. Furthermore, the complexes show an intraligand transition around 42000 $cm^{-1}$.

The values of the magnetic moments at room temperature of the copper complexes of the H3HF and $H_2$5,7DHF are in the normal range for Cu(II) complexes without metal-metal interaction (1.74-1.93 MB). The magnetic moment values at room temperature of Cu(7,8DHF)$(H_2O)_2$ and Cu(7,8DHF)(MeOH) are 1.43 and 1.40 MB respectively, suggesting metal-metal antiferromagnetic interaction. The complex Cu(7,8 DHF)$(NH_3)_2(H_2O)_3$ shows $\mu$ = 1.78 MB in the normal range for Cu(II) mononuclear complexes.

The polycrystalline EPR spectra of the complexes of copper at room temperature are axial. The simulation[28] of the EPR spectrum of the $Cu(3HF)_2$ complex show $g_{\parallel}$ = 2.25 and $g_{\perp}$ = 2.07 and that of $Cu(3HF)_2(NH_3)(H_2O)$ complex are 2.25 and 2.08, suggesting that the unpaired electron must be in $d_{x2-y2}$ (or $d_{xy}$) orbital. The EPR spectrum of the $Cu(H5,7DHF)_2(MeOH)_2$ complex is axial with a weak hyperfine coupling. The EPR parameters are $g_{\parallel}$ = 2.16 and $g_{\perp}$ = 2.04, while the value of the parallel hyperfine coupling constant $A_{\parallel}$ is about $160.10^{-4}$ $cm^{-1}$. The value of $g_{\parallel}$ / $A_{\parallel}$ quotient of 135 permits us to deduce an distorted octahedral environment around the Cu(II) ion.[29] Similar EPR parameters can be deduced from the EPR spectrum of the obtained $Cu(H5,7DHF)_2$ $(H_2O)_2$ complex ($g_{\parallel}$ = 2.22, $g_{\perp}$ = 2.03 and $A_{\parallel}$ = $166.10^{-4}$ $cm^{-1}$). The EPR spectrum of Cu(7,8DHF)$(NH_3)_2(H_2O)_3$ is rhombic with $g_z \approx 2.47$ ; $g_y$ = 2.10 and $g_3$ = 2.0. The EPR spectra of the Cu(7,8DHF)$(H_2O)_2$ and Cu(7,8DHF)(MeOH) are practically silent.

*Cleavage of pBR322 by Cu(7,8DHF)$(NH_3)_2(H_2O)_3$, Cu(7,8DHF)$(H_2O)_2$ and Cu(7,8DHF)(MeOH) complexes.-*

The addition of Cu(7,8DHF)$(NH_3)_2(H_2O)_3$ to pBR322 which is initially mostly supercoiled Form I, in the presence of a 100 fold excess of $H_2O_2$ causes cleavage of the plasmid to give both nicked (single strand break, ssb, Form II) and linear (double strand break, dsb, Form III) products. Figure 2 shows the progression of the cleavage with increasing complex concentration, in the presence of $H_2O_2$ (pH=8 ; T = 25°C). From this figure we can appreciate that a final concentration of 50 or 75 μM of the complex gives optimum cleavage in the presence of 5000 and 7500 μM $H_2O_2$ respectively, under this conditions, to give both nicked and linear products. Above this concentration, linear products are further degraded as indicated by the smear on the gel.

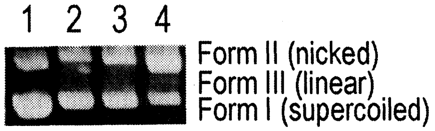

Figure 2. Cleavage of pBR322 in the presence of Cu(7,8DHF)$(NH_3)_2(H_2O)_3$ complex and $H_2O_2$. Lane 1, pBR322 control; lane 2, Cu(7,8DHF)$(NH_3)_2(H_2O)_3$ complex 10μM + $H_2O_2$ 1mM ; lane 3, Cu(7,8DHF)$(NH_3)_2(H_2O)_3$ complex 50μM + $H_2O_2$ 5mM ; lane 4, Cu(7,8DHF)$(NH_3)_2(H_2O)_3$ complex 75μM + $H_2O_2$ 7.5 mM.

The progression of the cleavage reaction with MPA (mercaptopropionic acid) was also examined. Figure 3 shows that the complex is a chemical nuclease at 50 and 75 μM, but as we compare with that of $H_2O_2$ we can deduce a lower nuclease activity of the Cu(7,8DHF)$(NH_3)_2(H_2O)_3$ complex in the presence of MPA.

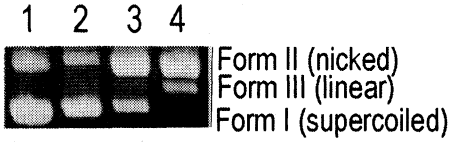

Figure 3. Cleavage of pBR322 in the presence of Cu(7,8DHF)$(NH_3)_2(H_2O)_3$ complex and MPA. Lane 1, pBR322 control; lane 2, Cu(7,8DHF)$(NH_3)_2(H_2O)_3$ complex 10μM + MPA 1mM ; lane 3, Cu(7,8DHF)$(NH_3)_2(H_2O)_3$ complex 50μM + MPA 5mM ; lane 4, Cu(7,8DHF)$(NH_3)_2(H_2O)_3$ complex 75μM + MPA 7.5 mM.

We tried to study the cleavage reaction of pBR322 in the presence of the other complex, Cu(7,8DHF)$(H_2O)_2$ in the same conditions as reported above. Figure 4 confirms the chemical nuclease activity of this complex in the presence of $H_2O_2$ or MPA or ascorbic acid. From this figure we can observe that the complex only shows nicked and linear forms in the complex concentration range from 20 to 40 μM. If we compare its nuclease activity in presence of $H_2O_2$ or MPA or ascorbic acid, we can deduce that the increasing activity order is : ascorbic acid > $H_2O_2$ > MPA.

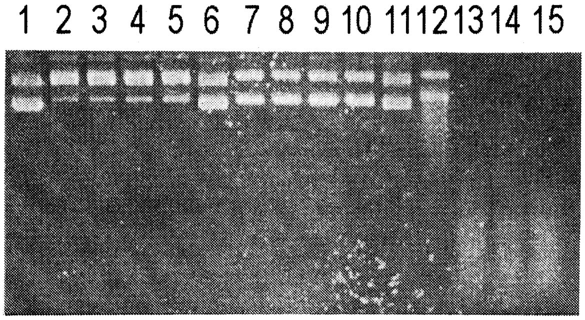

Figure 4. Cleavage of pBR322 in the presence of Cu(7,8DHF)$(H_2O)_2$ complex and $H_2O_2$, MPA and ascorbic acid(Asc). Lanes 1, 6 and 11 pBR322 control; lane 2, Cu(7,8DHF)$(H_2O)_2$ complex 20μM + $H_2O_2$ 2.0mM ; lane 3, Cu(7,8DHF)$(H_2O)_2$ complex 25μM + $H_2O_2$ 2.5mM ; lane 4, Cu(7,8DHF)$(H_2O)_2$ complex 30μM + $H_2O_2$ 3.0 mM; lane 5, Cu(7,8DHF)$(H_2O)_2$ complex 40μM + $H_2O_2$ 4.0mM; lane 7, Cu(7,8DHF)$(H_2O)_2$ complex 20μM + MPA 2.0mM ; lane 8, Cu(7,8DHF)$(H_2O)_2$ complex 25 μM + MPA 2.5mM ; lane 9, Cu(7,8DHF)$(H_2O)_2$ complex 30 μM + MPA 3.0mM ; lane 10, Cu(7,8DHF)$(H_2O)_2$ complex 40μM + MPA 4.0mM ; lane 12, Cu(7,8DHF)$(H_2O)_2$ complex 20μM + Asc 2.0mM ; lane 13, Cu(7,8DHF)$(H_2O)_2$ complex 25μM + Asc 2.5mM; lane 14, Cu(7,8DHF)$(H_2O)_2$ complex 30 μM + Asc 3.0 mM ; lane 15, Cu(7,8DHF)$(H_2O)_2$ complex 40μM + asc 4.0 mM. Reactions were carried out with 0.025μg/μl pBR322 at pH = 8 (Tris buffer), 25°C for 30 min.

Several control reactions have been carried out to insure that the Cu(7,8DHF)(MeOH) complex is responsible for the observed cleavage of pBR322. The amounts of DNA reaction mixtures containing $H_2O_2$, Cu(II) salts or Cu(7,8DHF)(MeOH) complex (or combinations thereof) are shown in Figure 5. From this we can conclude that the Cu(II) salts at concentrations between 20 to 40 μM do not cause the cleavage of the plasmid. When we observe the lanes 6 and 9 corresponding to a mixtures of Cu(II) salt and $H_27,8DHF$ (lane 6 with a 30 μM and lane 9, 40 μM in Cu(II) and the flavone respectively) we can appreciate that the cleavage reaction of DNA takes place showing Form I and Form II at different intensities, while when the concentration is 20 μM (lane 3) the figure presents an electrophoretic way similar to that of the pattern as consequence there are not nuclease activity. Lanes 4, 7 and 10 represent the cleavage reaction of pBR322 in presence of the Cu(7,8DHF)(MeOH) at 20, 30 and 40 μM. From lane 4 is clearly shown the nuclease activity of the complex, increasing their activity when the concentration increases in the lanes 7 and 10. If we compare lanes 3, 6 and 9 with lanes 4, 7 and 10, we can conclude that the complex has a more importnat nuclease activity than the metal ion, the ligand or a mixture of both.

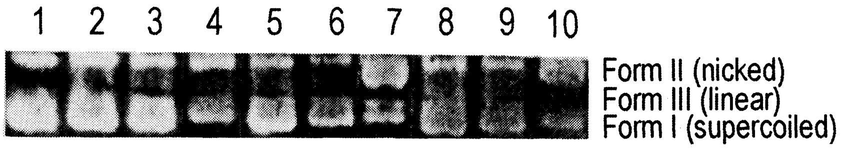

Figure 5. Cleavage of pBR322 by Cu(7,8DHF)(MeOH) in the presence of $H_2O_2$. Lanes (1) untreated pBR322 ; (2) with added 20μM Cu(II) salt and 2 mM $H_2O$ ; (3) with added 20μM [Cu(II) salt + 7,8DHF] and 2 mM $H_2O_2$; (4) with added 20μM of the Cu(7,8DHF)(MeOH) complex and 2mM $H_2O_2$ ; (5) 30 μM Cu(II) salt and 3 mM $H_2O_2$ ; (6) 30 μM [Cu(II) salt + 7,8DHF] and 3 mM $H_2O_2$ ; (7) 30 μM of the Cu(7,8DHF)(MeOH) complex and 3 mM $H_2O_2$ ; (8) 40 μM Cu(II) salt and 4 mM $H_2O_2$. (9) 40 μM [Cu(II) salt and 7,8DHF] and 4 mM $H_2O_2$ ; (10) 40 μM of the Cu(7,8DHF)(MeOH) complex and 4 mM $H_2O_2$ . Reactions were carried out with 0.025 μg/μl pBR322 at pH = 8.0 (Tris buffer), 25°C. for 30 min.

In order to assess some specificity, supercoiled pUCI plasmid DNAwas cleaved with the Cu(7,8DHF)MeOH, followed by probing with EcoRI. (Figure 6). When we compare lanes 1 and 2 that correspond to a result of the cleavage in the presence of the complex (lane 1) and afterwards digested with EcoRI (lane 2), the existence of three bands in the latter can suggest a preferential cleavage of the Cu(7,8DHF)(MeOH) complex.

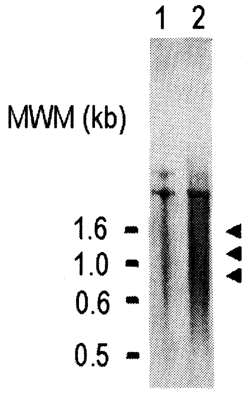

Figure 6. Cleavage of pUC18 by Cu(7,8DHF)(MeOH) complex in the presence of $H_2O_2$. Lanes (1) pUC18 treated with 50 μM of Cu(7,8DHF)(MeOH) complex and 50 mM of $H_2O_2$ ; (2) pUC18 treated with 50 μM of Cu(7,8DHF)(MeOH) complex and 50 mM of $H_2O_2$ and following cleavage with EcoRI.

From these experiences we can conclude that the $Cu(7,8DHF)(NH_3)_2(H_2O)_3$, $Cu(7,8DHF)(H_2O)_2$ and Cu(7,8DHF)(MeOH) complexes are chemical nucleases. We also plan to continue to study the possible specificity of the complex and the other related ones in the hope of discovering other potentially practical inorganic nucleases.

*Involvement of the Cu(I) in the reaction.-*

We employed bathocuproine as an agent that sequesters Cu(I) selectively. The Cu(I)-bathocuproine chelate has an absorption maximum at 480 nm. Under the conditions of our reaction, neither Cu(II) nor $H_27,8DHF$ interferes with the absorption maximum, whereas $H_27,8DHF$ and Cu(II) react to generate Cu(I) that in the presence of bathocuproine, gives rise to the maximum at 480 nm (Figure 7). The implication of these findings is that Cu(II) is reduced by $H_27,8DHF$, which does not establish whether Cu(I) is an endproduct or an intermediate in the reaction that occurs in the absence of sequestering compound. As we obtain the solid complexes, we think that the Cu(I) is an intermediate that it is oxidised to Cu(II) and reacts to $H_2$7,8DHF in order to form the complexes.

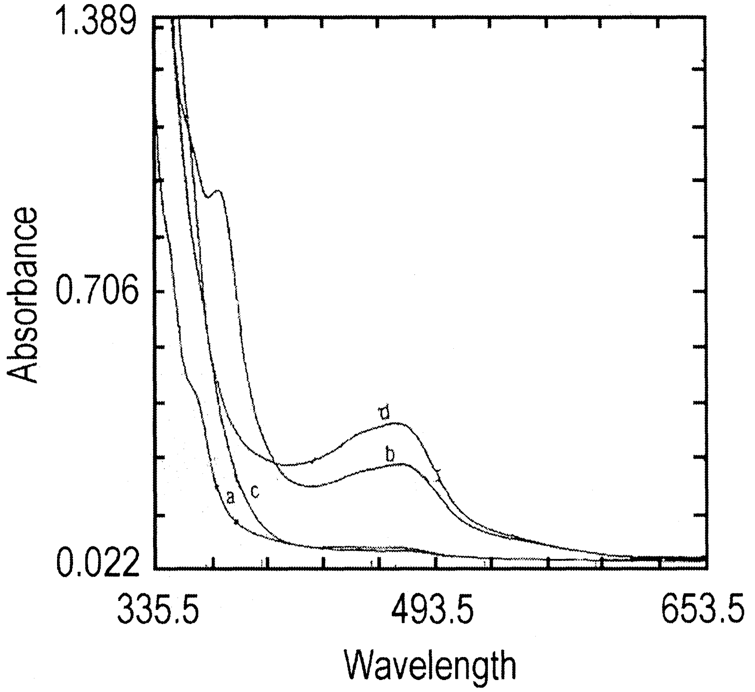

Figure 7. Detection of $H_2$7,8DHF-induced Cu(I) production by bathocuproine : a) Bathocuproine+Cu(II) 0.1mM ; b) Bathocuproine + Cu(I)0.02mM ; c) Bathocuproine + $H_2$7,8DHF 0.02mM ; d) Bathocuproine + $H_2$7,8DHF 0.02 mM + Cu (II) 0.04 mM. Bathocuproine was 0.1mM.

Our data establish that the copper complexes of $H_2$7,8DHF generate single-strand and double strand breaks in the presence of reducing agents such as ascorbic acid, $H_2O_2$ or MPA. These complexes have more nuclease activity than the mixtures of Cu(II) and $H_2$7,8DHF. If we observe the chemical formula of $H_2$7,8DHF, we can observe the presence of two OH contiguously. Hadi [11,12] reported the nuclease activity of the quercetin and other flavonoids in the presence of Cu(II) and oxygen and concluded that the presence of two OH groups in positions 3' and 4' of the B ring is required for this activity. Our results indicate that the two contiguous OH are necessary but they can occupy positions in the A ring also.

According the mechanisms proposed previously by Sigman[13] and Hadi [11,12] for copper nucleases, we suggest a similar one, where the flavone reduces the Cu(II) to Cu(I) after that the complex is intercalated to DNA and then reacts to $H_2O_2$ to give rise oxygen radical species and an oxidised form of $H_2$7,8DHF, that may be a group of compounds. The radicals are the responsible for the DNA cleavage.

**ACKNOWLEDGMENTS**

We greatly appreciate financial support from Comision Interministerial de Ciencia y tecnología (PM97-0105-C02-01) and the Generalitat Valenciana (GV-D-CN-09-145-96)

**REFERENCES**

1.PC. Hollman and MB. Katan, *Free Radic Res,* **1999**, Dec. 31, S75-80.
2. S. Hattori and I. Noguchi, *Nature*, **1959**, *184*, 1145.
3. G. Speir, V. Fülöp and L. Parkanyi, *Chem. Commun.*, **1990**, 512-513.
4. E. Balogh-Hergovich, G. Speier and G. Argay, *Chem. Commun.*, **1991**, 551-552.
5. E. Balogh-Hergovich, J. Kaizer and G. Speir, *Inorg. Chim. Acta*, **1997**, *256*, 9-14.
6. I. Lippai, G. Speier, G. Huttner and L. Zsolnai, *Acta Cryst.*, **1997**, *C53*, 1547-1549.
7. I. Lippai, G. Speier, G. Huttner and L. Zsolnai, *Chem. Commun.*, **1997**, 741-742.

8. E. Balogh-Hergovich, J. Kaizer and G. Speir, V. Fülöp and L. Parkanyi, *Inorg. Chem.*, **1999**, *38*, 3787-3795.
9. J. Pusz and M. Kopacz, *Pol. J. Chem.*, **1992**, *66*, 1935-1940.
10. J. Pusz and B. Nitka, *Microchemical J.*, **1997**, *56*, 373-381.
11. A. Rahman, F. Fazal, J. Greensill, K. Ainley, J.H. Parish and S.M. Hadi, *Molecular and Cellular Biochemistry*, **1992**, *III*, 3-9.
12. M. Said Ahmed, V. Ramesh, V. Nagaraja, J.H. Parish and S.M. Hadi, *Mutagenesis*, **1994**, *9*, 193-197.
13. D.S. Sigman, A.M. Mazundar and D.M. Perrin, *Chem. Revs.*, **1993**, *93*, 2295-2316.
14. K. Douglas, *Trans. Met. Chem.*, **1996**, *21*, 474-480.
15. S.T. Frey, H.H.J. Sun, N.N. Murthy and K.K. Karlin, *Inorg. Chim. Acta*, **1996**, *242*, 329-338.
16. F.V. Pamatong, C.A. Detmer III and J.R. Bocarsly, *J. Am. Chem. Soc.*, **1996**, *118*, 5339-5345.
17. C.A. Detmer III, F.V. Pamatong, and J.R. Bocarsly, *Inorg. Chem.*, **1996**, *35*, 6292-6298.
18. N. Ramirez-Ramirez, G. Mendoza-Diaz, F. Gutierrez-Corona and M. Pedraza-Reyes, *J. Biol. Inorg. Chem.*, **1998**, *3*, 188-194.
19. O. Baudoin, M.P. Teulade-Fichou, J.P. Vigneron and J.M. Lehn, *Chem. Commun.*, **1998**, 2349-2350.
20. S. Routier, V. Joanny, A. Zaparucha, H. Verin, J.P. Catteau, J.L. Bernier and C. Bailly, *J. Chem. Soc., Perkin Trans 2*, **1998**, 863-868.
21. T. Kobayashi, T. Okuno, T. Suzuki, M. Kunita, S. Ohba and Y. Nishida, *Polyhedron*, **1998**, *9*, 1553-1559.
22. C. Liu, J. Zhou, Q. Li, L. Wang, Z. Liao and H. Xu, *J. Inorg. Biochem.*, **1999**, *75*, 233-240.
23. A. Theodorou, M.A. Demertzis, D. Kovala-Demertzi, E.E. Lioliou, A.A. Pantazaki and D.A. Kyriakidis, *Biometals*, **1999**, *12*, 167-172.
24. H.J. Eppley, S.M. Lato, A.D. Ellington and J. M. Zaleski, *Chem. Commun.*, **1999**, 2405-2406.
25. I. Dubey, G. Pratviel and B. Meunier, *J. Chem. Soc., Perkin Trans. 1*, **2000**, 3088-3095.
26. S.M. Morehouse, H. Suliman, J. Haff and D. Nguyen, *Inorg. Chim. Acta*, **2000**, *297*, 411-416.
27. M.C. Montesinos, A. Ubeda, M.C. Terencio, M. Payá and M.J. Alcaraz, *Z. für Naturforch.*, **1995**, *50c*, 552-560.
28. WINEPR-Simfonia 1.25, Bruker Analytik GmbH, Kalsruhe, FRG, 199-1996.
29. U. Sakaguchi and A.W. Addison, *J. Chem. Soc. Dalton Trans.*, **1979**, 600-605.

**Received: February 21, 2001 – Accepted: February 28, 2001 –**
**Received in revised camera-ready format: March 1, 2001**

